# A dynamic perfusion based blood-brain barrier model for cytotoxicity testing and drug permeation

**DOI:** 10.1038/s41598-020-60689-w

**Published:** 2020-03-02

**Authors:** Basma Elbakary, Raj K. S. Badhan

**Affiliations:** 1 Applied Health Research Group, Aston Pharmacy School, Aston University, Birmingham, B4 7ET United Kingdom; 2 Aston Pharmacy School, Aston University, Birmingham, B4 7ET United Kingdom

**Keywords:** Cell growth, Blood-brain barrier

## Abstract

The blood-brain barrier (BBB) serves to protect and regulate the CNS microenvironment. The development of an *in-vitro* mimic of the BBB requires recapitulating the correct phenotype of the *in-vivo* BBB, particularly for drug permeation studies. However the majority of widely used BBB models demonstrate low transendothelial electrical resistance (TEER) and poor BBB phenotype. The application of shear stress is known to enhance tight junction formation and hence improve the barrier function. We utilised a high TEER primary porcine brain microvascular endothelial cell (PBMEC) culture to assess the impact of shear stress on barrier formation using the Kirkstall QuasiVivo 600 (QV600) multi-chamber perfusion system. The application of shear stress resulted in a reorientation and enhancement of tight junction formation on both coverslip and permeable inserts, in addition to enhancing and maintaining TEER for longer, when compared to static conditions. Furthermore, the functional consequences of this was demonstrated with the reduction in flux of mitoxantrone across PBMEC monolayers. The QV600 perfusion system may service as a viable tool to enhance and maintain the high TEER PBMEC system for use in *in-vitro* BBB models.

## Introduction

The blood-brain barrier (BBB) represents an insidious barrier for the delivery of therapeutic agents for a wide range of central nervous system (CNS) disorders. Penetration of the restrictive brain microvascular endothelial cell barrier is often hindered by the presence of a network of intra-cellular tight junction proteins, in addition to a network of membrane localised active transporter proteins and enzymatic metabolism processes.

The development and maintenance of an appropriate restrictive *in-vitro* BBB model is critical when assessing the potential for small molecule transport. Despite the rise in the use of *in-vivo* models for assessing BBB structure and function, *in-vitro* models are still widely used and have been developed from a range of species. However, a consensus on the most appropriate cellular system has still not been achieved, particularly in the context of assessing drug permeation and inherent barrier properties. For example, the human immortalised hCMEC/D3 cell, when grown in co-culture with astrocytes, yields low TEER values of approximately 140 Ω.cm^2^ ^1^, and those from primary endothelial cells from rodents yield TEER values of approximately 300 Ω.cm^2^ ^2^. Higher TEER values have been obtained with stem cell based systems (iPSC-derived endothelial cells) and neuronal progenitor cells, when exposed to chemical treatment, resulting in values of 3000–4000 Ω.cm^2^ ^3^ to promote BBB formation, although these often require specialised and costly methods to culture.

Although human brain tissue derived *in-vitro* BBB models are idealised for BBB studies, the lack of appropriate monolayer formation and reproductively, *in-vitro*, has led to other cellular models being attractive options. The use of a porcine primary cell culture system (PBMEC) reporting high TEER without the need for co-culture with astrocytes^4–6^, are a potentially viable high purity, high resistance and reproducible *in-vitro* blood brain barrier model.

However, a critical feature of the BBB endothelial missing in many current *in-vitro* models, is the exposure of cells to a laminar shear stress, a key mechanical force which alters cellular morphology and differentiation when compared to non-shear stress conditions and which is important in stimulating a stable BBB phenotype^7–13^.

Physiological shear stresses can range from 4–30 dyne cm^2^ and 1–4 dyne cm^2^ in the venous circulation^14^. A range of approaches have been used to simulate shear stress across brain endothelial cells, from microfluidic systems^15,16^, to hollow fibre constructs^17^.

Recently, the use of a dedicated perfusion chamber systems (Kirkstall Quasi Vivo^®^^18^) capable of utilising permeable insert systems have gained traction as viable perfusion based models systems for *in-vitro* barrier systems^19,20^.

In the current study, we presented a novel approach to employ an *in-vitro* BBB model developed using a primary porcine brain microvascular endothelial cell culture model grown on routinely utilised permeable-inserts, where BBB phenotype was enhanced through the action of dynamic perfusion of cell culture medium. To this end we utilised the Kirkstall Quasi Vivo^®^ 600 system to: (i) identify an optimal shear stress capable of the sustained growth of PBMEC; (ii) assess resultant changes in cellular viability and cellular morphology and (iii) examine the impact of shear stress on the permeability of mitoxantrone across an *in-vitro* permeable-insert BBB model.

## Results

### Identification of optimal sheer stress

**Figure 1:**
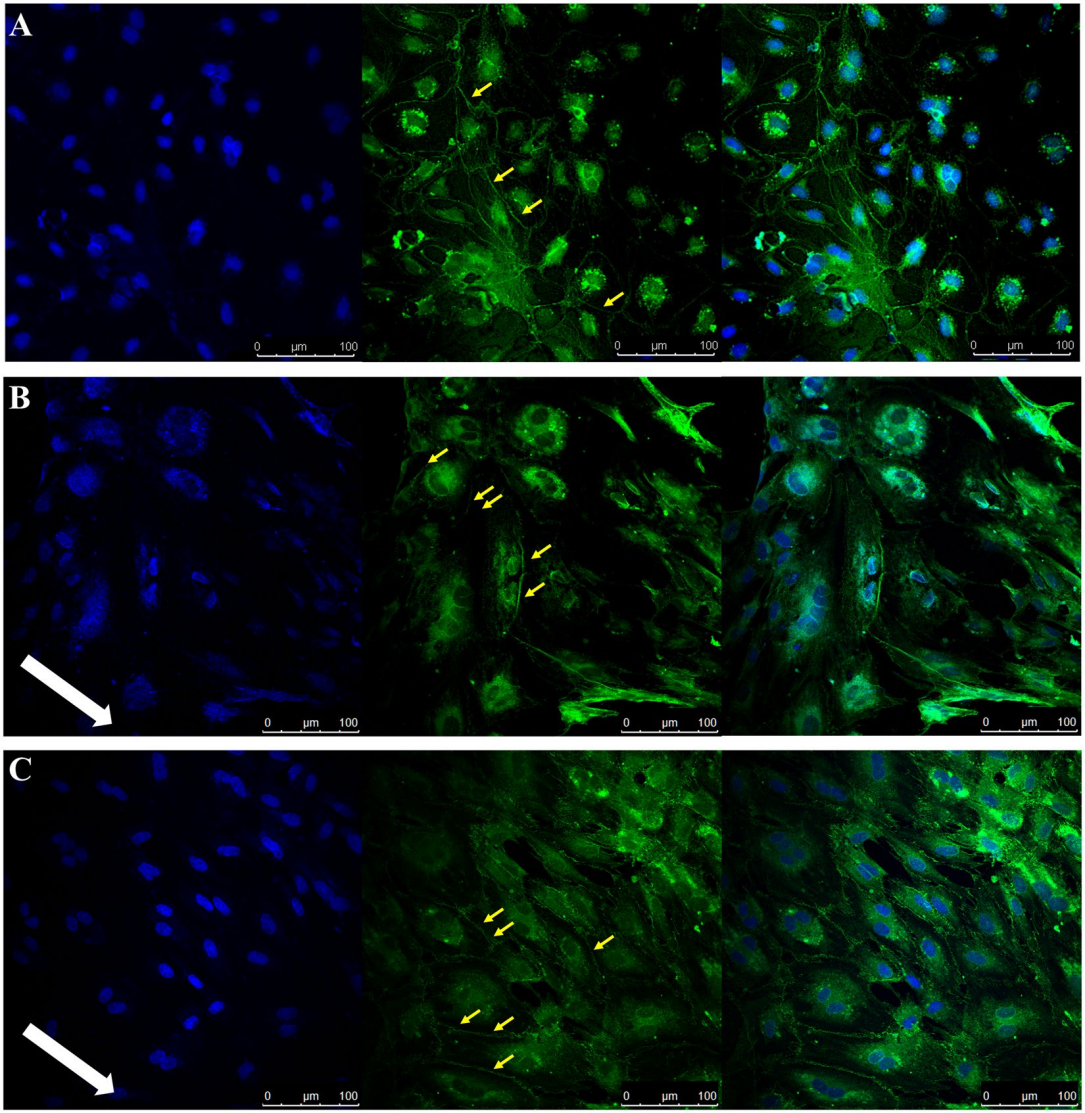
Immunocytochemistry images obtained following PBMEC staining for DAPI (left panel), ZO-1 (middle panel) and merged (right panel) when grown on bovine collagen (50 µg/mL) and fibronectin (7.5 µg/mL) coated coverslips. (**A**) PBMEC under static media conditions, (**B**) PBMEC grown under low flow (275 µL/min) and (**C**) PBMEC under high flow (550 µL/min) for 48 hours using the QV600. Images were taken using a Leica SP5 TCS II MP confocal microscope. White arrow indicates the direction of flow. Yellow arrows indicate formation of tight junctions.

In order to identify optimal sheer stress for use with PBMEC, coverslips were raised and exposed to low (275 µL/min) and high (550 µL/min) flow rates and compared to matching coverslips grown under static conditions and subsequently stained for ZO-1. Under static conditions, limited cell-to-cell ZO-1 formation is evident (Fig. 1A). When the flow rate was increased to low (Fig. 1B) and high (Fig. 1C), cellular reorganisation was evident with the cell-to-cell ZO-1 protein formation. However, the translocation of ZO-1 to the cytoplasm was also evident under low and high flow rates.

### Cellular viability under shear stress

**Figure 2:**
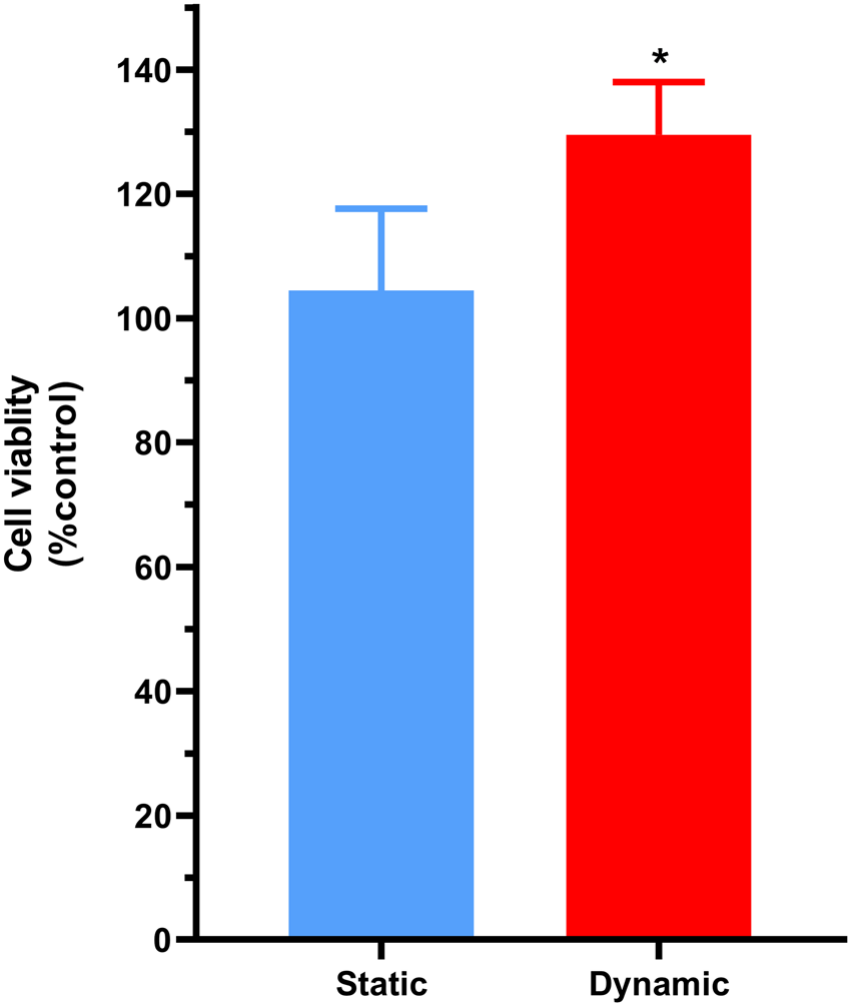
Cellular viability of PBMEC grown on coverslips under static media and dynamic high flow (550 µL/min) for 96 hours, using an MTT assay. Dynamic results were normalised to the mean of the static results. n = 9 coverslips in 3 independent experiment. *p ≤ 0.05.

To assess the impact of shear stress on PBMEC cellular viability, high flow (550 µL/min) was applied for 96 hours and cellular viability of PBMEC (grown on coverslips) assessed using an MTT cellular viability assay. The presence of high flow for 4 days did not reduce the viability of PBMEC, with a nominal, but significant (P < 0.05) increase in viability for dynamic shear stress (Fig. 2).

### Assessment of the impact of shear stress on the transendothelial electrical resistance of PBMEC

To investigate the formation of a high resistance barrier, the TEER from monolayers of ‘60 s’ grown on permeable inserts (24-well, 0.33 cm^2^) were determined in static conditions and under dynamic shear stress.

**Figure 3:**
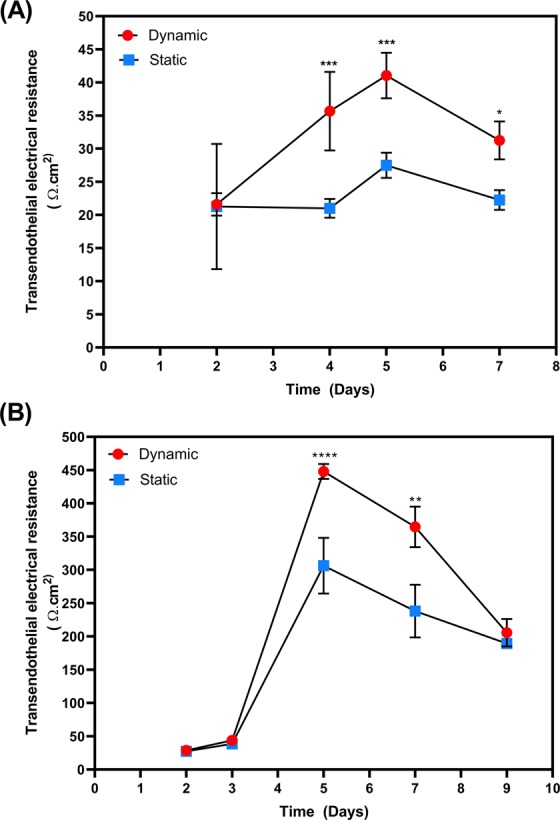
TEER measured following growth of PBMEC on permeable cell culture inserts (24-well, 0.33 cm^2^) under static and dynamic (550 µL/min) conditions. TEER of PBMEC were measured when grown on permeable inserts in the (**A**) absence and (**B**) presence of barrier forming additives. *p ≤ 0.05, **p ≤ 0.01, ***p ≤ 0.001 and ****p ≤ 0.0001. n = 12 in replicates of 3 in 4 independent experiments.

To assess whether shear stress is capable of inducing barrier formation, PBMEC were grown on permeable inserts and subjected to flow at 550 µL/min, without the addition of any barrier forming additives. At 4 days post-seeding, inserts exposed to flow demonstrated a significantly higher TEER (35.7 Ω.cm^2^ ± 5.1 Ω.cm^2^) compared by those maintained in static culture conditions (21 Ω.cm^2^ ± 1.5 Ω.cm^2^) (p ≤ 0.001), which was maintained through to day 7 post seeding (Fig. 3A).

To further assess the ability of shear stress to induce barrier formation, static and dynamic inserts were exposed to endothelial tight junction inducing agents on day 3 post seeding for 24 hours only. On day 4, TEER values significantly increased under both static media (to 306.3 Ω.cm^2^ ± 41.9 Ω.cm^2^) and dynamic flow (to 448.1 Ω.cm^2^ ± 11.3 Ω.cm^2^) (p ≤ 0.0001) which was maintained to day 7 (Fig. 3B).

### Tight junction formation and cellular reorganisation under shear stress

**Figure 4:**
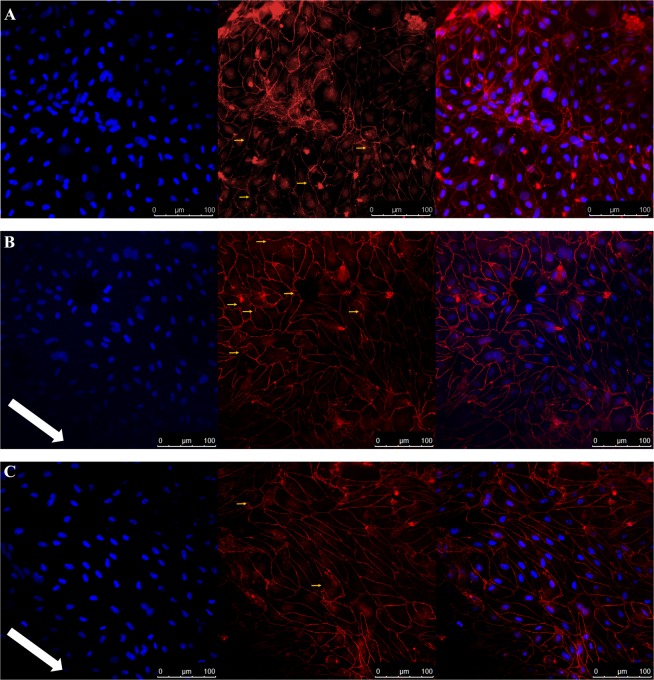
Immunocytochemisty images obtained following PBMEC staining for DAPI (left panel), ZO-1 (middle panel) and merged (right panel) when grown on permeable inserts. (**A**) PBMEC under static media conditions, (**B**) PBMEC grown under high flow (550 µL/min) for 24 hours and (**C**) PBMEC grown under high flow (550 µL/min) for 48 hours using the QV600. Images were taken using a Leica SP5 TCS II MP confocal microscope. White arrow indicates the direction of flow; yellow arrows indicate disrupted tight junction.

The expression of the tight junction protein ZO-1 was assessed using immunocytochemistry on permeable inserts maintained in either static media or exposed to shear stress (550 µL/min). In the absence (Fig. 4A) and presence (Fig. 4B,C) of shear stress, cellular labelling with ZO-1 antibody demonstrated the ability of PBMEC to form tight junctions. Under static conditions (Fig. 4A), the cellular morphology was indiscriminately organised with a discontinuous serrated patters. However, under shear stress, linear realignment of the PBMEC was particularly visible following 24-hours (Fig. 4B) and 48-hours (Fig. 4C) of shear stress exposure, and was largely localised to the intracellular junctions of cells, with limited discontinuous tight junction formation.

**Figure 5:**
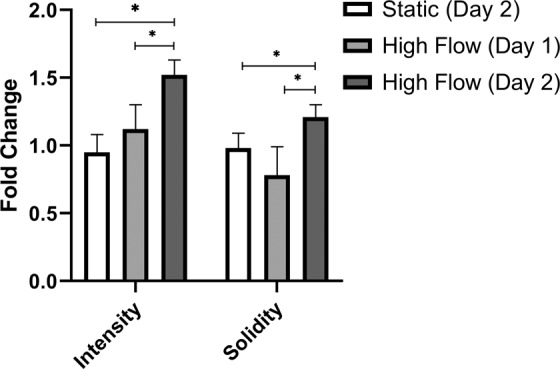
ZO-1 junctional fluorescence intensity and solidity as quantified by junctional regions and when normalised to static controls. *p ≤ 0.05.

Furthermore, the ZO-1 junctional intensity was significantly higher following 48-hours exposure to flow (1.52 ± 0.11 fold) than in static control (p ≤ 0.05). In addition, there was a statistically significant greater junctional intensity at 48-hours when compared to 24-hours exposure (1.12 ± 0.18 fold) (p ≤ 0.05) (Fig. 5). Junctional solidity was also significantly greater at 48-hours (1.21 ± 0.09 fold) (p ≤ 0.05) when compared to static conditions, with a statistically significant decrease in solidity at 24-hours (0.78 ± 0.21 fold) (p ≤ 0.05) when compared to 48-hours (Fig. 5).

### Mitoxantrone permeability across PBEMC

**Figure 6:**
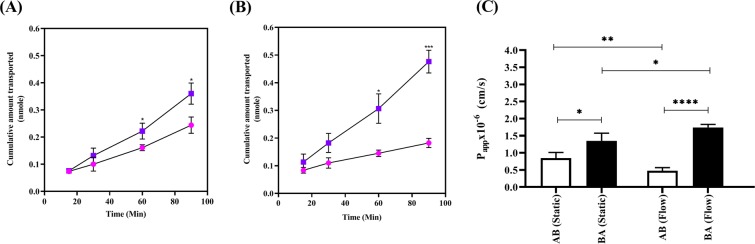
Mitoxantrone flux across PBMEC grown on permeable inserts. Mitoxantrone transport in apical-to-basolateral (AB) (circles) or basolateral-to-apical (BA) (squares) under (**A**) static media or (**B**) when exposed to high flow (550 µL/min) for 48 hours with associated apparent membrane permeability (P_app_) values in the AB or BA directions (**C**). *p ≤ 0.05, **p ≤ 0.01, ***p ≤ 0.001 and ****p ≤ 0.0001. n = 16 for static and dynamic, in 4 independent experiments.

To demonstrate the functional impact of dynamic media flow on BBB properties, the permeability of the antineoplastic agent mitoxantrone was assessed across PBMEC grown on permeable inserts, in the absence (Fig. 6A) and presence of flow (Fig. 6B).

When compared to static conditions, the impact of shear stress resulted in a significant increase in the cumulative amount transported in the BA direction (Fig. 6B) when compared to the AB direction (Fig. 6A). The apparent membrane permeability (P_app_) in the absence of flow (P_app,AB_: 0.84 × 10^−6^ cm/s ± 0.16 × 10^−6^ cm/s and P_app,BA_: 1.35 × 10^−6^ cm/s ± 0.23 × 10^−6^ cm/s) resulted in an efflux ratio of 1.6. However, under the exposure of flow, there was a significant increase in efflux ratio to 3.6 with a significantly reduced A-to-B flux (p ≤ 0.01) and a significant increase in B-to-A flux (p ≤ 0.05) (P_app,AB_: 0.48 × 10^−6^ cm/s ± 0.09 × 10^−6^ cm/s and P_app,BA_: 1.74 × 10^−6^ cm/s ± 0.08 × 10^−6^ cm/s).

## Discussion

The development and maintenance of an *in-vitro* BBB model is critical when assessing the potential for small molecule transport, with the ability to form a coherent and robust barrier being paramount. Critical to the establishment of models, is the maintenance of appropriate *in-vitro* culturing conditions.

A key component to the formation of the BBB *in-vivo*, is the presence of circumferential stress on the walls of the brain microvascularate, which plays an important role in the morphology and functional capacity of endothelial cells, along with governing signalling and transport processes within the neurovascular unit^9–13^. The average shear stress within the arterial circulation is 4–30 dyne cm^2^ and 1–4 dyne cm^2^ in the venous circulation^14^. BBB models which have incorporated laminar shear stress have demonstrated the lowest permeability to sucrose and mannitol tracers, highlighting the critical role that laminar shear plays in stimulating a stable BBB phenotype^7,8^.

However, there is currently no consensus on an appropriate human BBB *in-vitro* model, particularly with regards to an appropriate cell type or culturing method, when used for drug permeation and inherent barrier properties. For example, the human immortalised hCMEC/D3 cell, when grown in co-culture with astrocytes, yields low TEER values of 140 Ω.cm^2^^1^, and those from primary endothelial cells from rodents yield TEER values of approximately 300 Ω.cm^2^^2^. Higher TEER values have been obtained with stem cell based systems (iPSC-derived endothelial cells) and neuronal progenitor cells, when exposed to chemical treatment, resulting in values of 3000–4000 Ω.cm^2^ ^3^ to promote BBB formation

Given the low TEER or complexity in culturing approaches, we adopted the use of a porcine primary immortalised cell culture system (PBMEC) reporting high TEER without the need for co-culture with astrocytes^4–6^.

In the present study we applied the PBMEC model system^4^ within the Kirkstall Quasi Vivo^18^ interconnected chambers system (QV600), which can accommodate both cells grown on coverslips (13 mm diameter) and permeable inserts (24-well plate, 13 mm diameter) in an attempt to determine whether the impact of localised perfusion on PBMEC would enhance barrier formation, as measured by the TEER^4,21,22^. The presence of hemodynamic shear stress is an important element within endothelial cells which is absent in cells cultured on inserts under static conditions, and contributes to the polarisation of the brain endothelium structure as well as governing the expression and localised of drug transporter systems.

The QV600 chambers are connected in series or parallel, with perfusion within all chambers from a central perfusion pump. The key advantage is the rapid perfusion of media and oxygen transport. Whist the QuasiVivo system has been used by other groups to assess the benefit of dynamic media flow within an interconnected system of chambers with different cell cultures^19^, its use with BBB cell types grown on permeable inserts has been limited, particularly with high TEER primary origin cell lines.

Furthermore, whilst the use of human origin cells in microfluidic systems has gained traction, these remain a novel and niche research tool^15,16^ which often fail to represent macroscale environments and make it difficult for research with established techniques, e.g. permeable insert barrier systems, to easily adopt such systems^23^.

**Figure 7:**
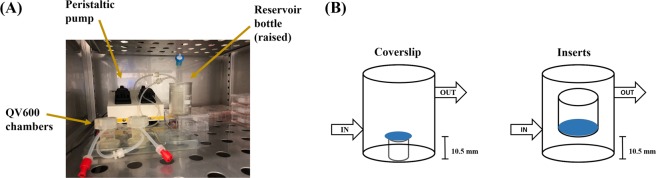
A diagrammatical representation of: (**A**) the setup of the QV600 and (**B**) the location of coverslips in comparison to the use of permeable inserts.

We first aimed to assess the impact of shear stress on PBMEC when grown on coverslips and evaluated both changes in cellular morphology along with cellular viability. The location of the coverslip within the QV600 chamber is important when assessing the impact of sheer stress on cellular viability, given that there is at least a 200–300-fold decrease in sheer stress when approaching the base of the chamber^18^. Furthermore, the use of permeable inserts to develop a BBB monolayer places the cell layer within the vicinity of the chamber inlet/outlet^24^. To this end, PBMEC were seeded on coverslips and raised to the equivalent height of the filter membrane of the permeable inserts (Fig. 7), prior to exposure to low (275 µL/min) and high (550 µL/min) flow rates.

The impact of sheer stress on PBMEC morphology was evident at both low and high flow rates, with significant reorientation under flow in addition to improved clarity of cell-to-cell tight junction formation (Fig. 1B,C) when compared to cells grown under static conditions (Fig. 1A).

Given that PBMEC have not been previously used within the QV system, the present study attempted to first identify an appropriate flow rate for use within PBMEC. Drawing on work conducted previously by Miranda-Azpiazu *et al*.^19^, we selected a flow rate of 550 µL/min to assess cellular viability. Miranda-Azpiazu *et al*.^19^ utilised a maximum flow rate of 300 µL/min and suggested higher flow rates are sustainable for endothelial cells within the same perfusion-based system we used. Furthermore, the flow rates chosen were within the range suggested to ensure laminar flow within the perfusion system^18^. This would mimic the haemodynamic flow and forces within the broad linear regions of the endothelial structure as opposed to the branched regions where non-linear/disturbed flow would be more apparent^25^. Given that morphological alterations were evident under both low and high flow rates (Fig. 1), we focussed upon the high flow rate for a comparison of cellular viability between static and dynamic conditions.

This higher flow rate did not adversely affect cellular viability, but rather resulted in a significant (p = 0.031) 28.2% increase in viability when exposed for 4 days to high flow (Fig. 2). The increase in viability reported herein was not to the same extent as those reported by Mazzei *et al*.^18^, >50% increase, whom utilised astrocytes cultured on coverslips and exposed to flow within the QV500 system without raising the height of the coverslips.

Having established that higher flow rates are capable of sustaining the growth of PBMEC within the QV600 system, we next wanted to assess the impact of sheer stress on barrier formulation when PBEMC were grown on permeable inserts. A benefit of the PBMEC system is the reproducible nature of the high TEER small vessels isolated from fresh porcine hemispheres (termed ‘60 s’)^4–6^, which can reach in excess of 800 Ω.cm^2^ (in 1.1 cm^2^, 12-well permeable inserts), when compared to the lower TEER hCMEC/D.3, rodent and iPS derived systems. The PBMEC system does not require co-culturing cells with astrocytes, rather the use of astrocyte-conditioned media (ACM) in a 50% ratio with PBMEC media is sufficient to aid in the establishment of a robust BBB, with the supplement within tight junction formation enhancers (250 µM cAMP, 17.5 µM RO 20–1724 and 550 nM hydrocortisone).

In the absence of barrier forming additives, sheer stress resulted in a statistically significant (p < 0.05) increase in TEER throughout the study period of 7 days, when compared to inserts grown under static conditions (Fig. 3A). A peak TEER of 35.7 Ω.cm^2^ ± 5.1 Ω.cm^2^ was reported compared to that of static, 21 Ω.cm^2^ ± 1.5 Ω.cm^2^ (p ≤ 0.001) highlighting the positive impact shear stress had on barrier formation, despite the absence of media supplementation.

With the addition of supplementation, we noted a 46% increase in peak TEER under shear stress when compared to the absence, with TEER under shear stress significantly higher from day 5 onwards (Fig. 3B). The peak TEER under flow, 448.1 Ω.cm^2^ ± 11.3 Ω.cm^2^, is higher than those conducted in other cells lines^1–3^. However, it should be noted that the QV600 accepts only 24-well multiplate inserts with a surface area of 0.33 cm^2^. PBMEC established by other groups typically utilise 12-well multiplate inserts (1.1 cm^2^)^4,5^.

In the QV600, the insert is used in such a way that the flow of media attempts to mimic human brain interstitial flow, reported to be <500 uL/min in human brain^26,27^. The role of sheer stress under these conditions are difficult to conceptualise. However, it is known that shear stress can exert a mechanism strain and pressure effect on cells, impacting upon cell differentiation and growth^28–30^. With flow in the basolateral side of the membrane, the porous nature of the membrane would allow this pressure differential, which would enhance both the transfer of small molecules such as oxygen and CO_2_ in addition to providing physical forces to the cell monolayer^31^. This is critical as increased dissolved CO_2_ concentrations^32,33^ can reduce the intracellular pH and thereby affect cell metabolism^34^, in addition to high levels of dissolved CO_2_ reducing cellular growth rates^35,36^.

Furthermore, the increase in TEER may also be a direct result of the rearrangement of cellular morphology on the cells, which is commonplace and the defining feature of laminar flow within the vascular network. For example, within athlorsclerotic-prone regions of the endothelia, there is a strong correlation with the failure of endothelial cells to elongate and align^37–42^. A similar increase in TEER was noted by others when using the QV600 to develop an *in-vitro* pulmonary model cultured on permeable inserts^20^.

To examine morphological changes under flow, permeable inserts were subsequently assessed for the formation and localisation of tight junction protein marker ZO-1. Under static conditions, the localisation of ZO-1 was presented with a discontinuous pattern of formation (Fig. 4A). In the presence of shear stress, clear realignment of the cells was evident, with more pronounced TJ formation after 48-hours (Fig. 4C) rather than 24-hours (Fig. 4B) of exposure of shear stress. The fluorescent intensity of the junction protein expression was further assessed and demonstrated that 48 hours of exposure yielded statistically significant (p ≤ 0.05) higher levels of ZO-1 when compared to static PBMEC (Fig. 5). Further, the solidity of the tight junctions were greater at 48-hours compared to 24-hours when compared to static conditions (p ≤ 0.05). Although the decrease in solidity at 24-hours, may be a result of realignment of junctional morphology (Fig. 4). This increasing expression of tight junction markers has been previously demonstrated, albeit it under higher shear stress in alternative hollow-system bioreactors^13,43^, and in QV500.

In the scope of developing a BBB, the use of permeable inserts allow for the monitoring of monolayer formation and small molecular transport. To assess the impact of shear stress on these processes, we assess the permeation of the antineoplastic agent Mitoxantrone across PBMEC grown under static and shear stress-exposed inserts. Mitoxantrone undergoes active transport partly through *ACBG2* and *ABCB1*^44,45^ and is classified as a low permeability compound^46^.

Under static conditions, the resultant apparent permeability in the BA direction, when compared to the AB direction, (i.e. the efflux ratio) was 1.6-fold greater and statistically significant (p < 0.05) suggesting an active efflux process (Fig. 6A). However, in the presence of shear stress, this increased to 3.6-fold (Fig. 6B) with a concomitant reduction in P_app,AB_ suggesting a tighter barrier formation limiting mitoxantrone flux (Fig. 6C).

In conclusion, for the first time we have demonstrated the impact of the novel application of sheer stress to an easy to isolate, cost-effective and high reproducible TEER BBB model derived from porcine brain microvascular cells (PBMEC) when grown on routinely utilised permeable inserts culturing system. The use of this well-established culturing approach, provides an ability to incorporate, with ease, BBB *in-vitro* models into the commercially available QuasiVivo® perfusion system platform without the necessary complications of other perfusion-based systems, such as microfluidic platforms. Microfluidic systems have gained some traction but still remain a niche research tool for perfusion based cell culture systems, given that the vast majority of research groups working on barrier models are still using permeable-inserts (e.g. Transwell®) systems^47–51^ for assessment of the impact of perfusion on the functional activity of barrier due to commercial availability, high-throughput potential and ease of use. Several well documented challenges and limitations have been reported with microfluidics systems, for example the lack of standardised parameters and critical experiment factors such as: (i) the use of an appropriate shear stress; (ii) defining an appropriate TEER cut-off for monolayer formation, which are typically low (<250 Ω.cm^2^)^52,53^ and (iii) appropriate application of paracellular permeability markers^54–57^. These limitations make comparison to well established BBB models (e.g. permeable insert models) difficult. Further, the well cited scalability issues and specialised microfluidics fabrication equipment severely limits the use and validation of such models across the wider scientific community^54^. In comparison, the proposed model system we implemented is commercially available and well validated, utilising existing and well-established permeable insert systems for barrier formation. Further, using a simplistic PBMEC system from porcine hemispheres, we were able to demonstrate significantly higher TEER in tradition inserts in addition to enhanced TEER using a commercially available perfusion system, with the benefits of being reproducible and requiring little technical knowledge when compared to microfluidic systems.

The novelty here-in is the fact that we have, for the first time, highlighted the application of the easy to isolate and cost-effective PBMEC BBB model within the QuasiVivo® system, which for the first time demonstrated a resultant impact on TEER and BBB phenotype enhancement. Further, we demonstrated higher TEER values in the absence or presence of perfusion, when compared to other microfluidic systems employing rodent or human derived BBB models, alongside further demonstrated the functional consequence of shear stress on small-molecule transport.

Our results highlight an apparent change in both cellular morphology and enhanced barrier formation, providing a valuable tool to assess both the neurotoxicity of molecules at the BBB but also their permeability across widely utilised permeable insert based BBB monolayer systems.

## Methods

### Isolation of porcine brain capillaries

The PBMEC model was developed previously^4,5^ and has been adapted in places for this study.

Porcine brain hemispheres were acquired from a local abattoir (Long Compton, Oxford, UK) within 30 minutes of sacrificing. Brains were transported on ice to the laboratory, in a large sterile box containing Leibovitz-15 (L-15) media supplemented with 1% v/v penicillin and streptomycin.

The hemispheres were placed into a clean beaker containing PBS with 1% v/v penicillin and streptomycin and washed to remove the meninges, blood vessels, choroid plexus and capillaries within the sulci.

The white matter was carefully removed, and the grey matter excised and placed into a sterile beaker containing MEM supplemented with 10 mM HEPES and 1% v/v penicillin and streptomycin. The grey matter was chopped into smaller pieces using a sterile scalpel and passaged through a 50 mL syringe into a T75 flask containing 50 mL containing MEM supplemented with 10 mM HEPES and 1% v/v penicillin and streptomycin.

Thereafter 15 mL of the tissue suspension was transferred into a 40 mL Dounce tissue homogeniser containing 25 mL of MEM supplemented with 10 mM HEPES and 1% v/v penicillin and streptomycin. The tissue suspension was homogenised with a loose (“A”) pestle gently for 15 strokes, followed by 15 strokes using the tight (“B”) pestle. Thereafter, the tissue homogenates was transferred to a sterile T175 and the process repeated for the remaining tissue suspension.

The collected tissue homogenate was subsequently filtered through a 150 µm nylon mesh (Plastok Limited, UK) and the collected filtrate was filtered again through a 60 µm nylon mesh, with filters being retained separately. The filters were separately placed into a petri dish containing 80 mL of digest mix consisting of M199 media containing: trypsin (211 U/mg); DNase I (2108 U/mg); collagenase (223 U/mg) (Worthington Biochemical, NJ, USA); 10% v/v FCS and 1% v/v penicillin and streptomycin. Petri dishes were labelled as ‘150’s or ‘60 s’ to demark each filter size, and incubated in 37 °C for 1 hour at 150 rpm in an orbital shaker. Thereafter the filters were carefully washed, and the digest mix transferred to 50 mL centrifuge tubes and centrifuged at 4 °C for 5 minutes at 5000 rpm, before being suspended in fresh MEM supplemented with 10 mM HEPES and 1% v/v penicillin and streptomycin. This process was repeated three times before the pellets were resuspended in cryopreservation media (10% v/v DMSO and 90% w/v FCS) for long-term storage in liquid nitrogen and labelled as ‘150 s’ (suitable for gene/protein expression/functional activity) and ‘60 s’ (suitable for BBB model development).

### Development of a BBB model

A T75 flask was coated with bovine collagen (50 µg/mL) prior to addition of cells from a thawed ‘60 s’. Thawed cells were suspended full PBEC media (Phenol red free DMEM (ThermoFisher, UK), 10% v/v plasma derived bovine serum (PBDS) (Firstlink, UK), 1% v/v antibiotic-antimycotic, 1% v/v L-glutamine, 125 µg/mL heparin. Cells were grown for 2 days before the addition of 4 µg/mL puromycin 4 µg/mL for 2 days, in order to eradicate pericyte contamination. Thereafter, PBMEC cells were maintained in astrocyte conditioning media (ACM) collected from the growth of rat C6 astrocytes (Cell Line Services, Germany), in a 1:1 (PBEC:ACM), until confluence (8–10 days post seeding) and are referred to as passage 0. Following trypsinisation, PBMEC cells were subsequently seeded at a density of 3x10^4^ cells/cm^2^ into 0.33 cm^2^ permeable inserts (Greiner BioOne transparent ThinCerts® 24 well) coated with bovine collagen (50 µg/mL) and fibronectin (7.5 µg/mL) and maintained in PBMEC:ACM, and considered as passage 1.

Barrier integrity and formation was assessed through the determination of the transendothelial electrical resistance (TEER), which was measured every 2 days using a chop-stick electrode (World Precision Instruments STX2). Tight-junction formation was enhanced through the addition of 250 µM cAMP, 17.5 µM RO 20–1724 and 550 nM hydrocortisone and the use of phenol-red free DMEM in the absence of serum for 24-hours prior to the initiation of an assay, with TEER values used to assess barrier integrity.

### Setting up the Quasi Vivo 600

To simulate dynamic perfusion of media within the cell culturing environment, the Quasi-Vivo 600 (QV600) interconnected chamber system was utilised. This allows for the use of permeable inserts and coverslips within chambers. The QV600 consists of chambers, a media reservoir, a peristaltic pump and associated tubing connectors (Fig. 7A). In order to utilise permeable inserts, the media level within each chamber was raised by increasing the height of the reservoir bottle by 5 cm with luer locks sealing the upper chambers (Fig. 7B).

Prior to use, the QV600 system was sterilised by submerging in 70% v/v ethanol for 24 hours, followed by exposed to UV light for 6 hours and flushed for 1 hour with PBS supplemented with 1% v/v penicillin and streptomycin.

Each peristaltic pump provided two independent channels, which were used with 3 chambers each. To assess shear stress, we considered flow rates within a range of 0–600 µL/min (0–11.0 × 10^−6^ Pa or 0–110 × 10^−6^ dyne/cm^2^) or flow speed of 0–1.69 × 10^−6^ m/s, with sheer stress (Pa) and flow speed (m/s) as described by Miranda-Azpiazu *et al*.^19^.

### Determination of optimal flow rate

Circular cover slips (13 mm) were coated with bovine collagen (50 µg/mL) and fibronectin (7.5 µg/mL) prior to seeding with 30,000 cells (from passage 1) and grown in 24-well plates in PBMEC:ACM. On day 3, coverslips were carefully transferred to QV600 chambers and raised by 10.5 mm using an inverted standing insert (Millicell 12 mm insert), to match the height of the permeable insert membrane when seated within the QV600 chamber (Fig. 7B).

Following optimisation of initial flow rates, cells were subjected to flow at 275 µL/min and 550 µL/min for 48 hours. Thereafter, cell morphology was assessed using light microscopy followed by assessment of tight junction formation through immunocytochemistry. Coverslips were washed with ice cold PBS three times and fixed in 4% w/v paraformaldehyde for 10 minutes. The coverslips were then washed three times with PBS and cells permeabilised using 0.02% saponin for 10 minutes followed by a further cycle of washing in PBS three times.

Cells were then blocked with 6% v/v goat serum (Sigma, UK) for 5 hours, prior to incubation with the ZO-1 primary antibody (ThermoFisher, UK: ZO-1 1A12 monoclonal) prepared in blocking buffer at a 1:100 dilution overnight at 4 °C. Thereafter, the coverslips were washed three times with ice cold PBS followed by the addition of the secondary antibody (ThermoFisher, UK: 0.5 µg/ml goat anti-mouse IgG H + L superclonal secondary Alexa 488®) for 2 hours at room temperature. The cells were then washed with ice cold PBS three times and mounted on a microscope slides using Fluoroshield with DAPI (Sigma, UK).

TJ formation was subsequently assessed using an upright confocal microscope (Leica SP5 TCS II MP) and visualised with a 40× oil immersion objective. Images were acquired with a argon laser at 494 nm and a helium–neon laser to visualise DAPI at 461 nm.

The formation intensity and solidity of tight junctions were analysed using image quantification using Fuji^58^ based upon an adapted method previously reported by Terryn *et al*.^59^ and McNeil *et al*.^60^. In brief, a baseline threshold was determined prior to analysis. Thereafter, automated pixel quantification was conducted throughout the region of interest, using an inverted greyscale image process which yielded tight junctions as black on a white background. Junctional ‘intensity’ was reported as the pixel density relative to static control. For ‘solidity’, each image was analysed by reinverting the greyscale and quantifying the density of ‘white’ pixels within the TJ borders, with results reported relative to the static control.

### MTT cell viability assay

In order to assess the impact of shear stress on the viability of the cells, an 3-(4,5-dimethylthiazol-2-yl)-2,5-diphenyltetrazolium bromide (MTT) assay was conducted using PBMEC grown on cover slips that were subjected to laminar flow for 48 hours (at an optimal flow rate determined previously) (n = 6) and under static conditions (n = 6). Thereafter, inserts were washed once with warm PBS prior to being incubated with 0.5 mg/mL MTT for 4 hours at 37 °C and 5% CO_2_. The resulting formazan crystals were dissolved in DMSO (100 µL/insert) for 15 minutes, before being transferred to a clear 96 well plate and with the UV-absorbance of the formazan crystals measured at 570 nm (Tecan Spark 10 M).

### Dynamic 2D models

PBMEC grown in permeable inserts intended for use in the QV600 were first seeded into permeable inserts under static conditions within a 24-well plates and grown for 3 days prior to transfer into the QV600 chambers (n = 20).

An acceptable monolayer formation under static and shear stress was determined using the TEER value (EVOM, World Precision Instruments, USA) and corrected for background resistance (coated inserts without cells) and by the surface area of the insert (0.33 cm^2^). In addition, the paracellular marker Lucifer yellow (LY) was used to confirm appropriate monolayer formation with a criterion of <1% LY permeation across monolayers deemed acceptable. LY permeability was assessed by the addition of 100 µM LY (Sigma, UK) prepared in HBSS into the apical chamber for 60 minutes at 37 °C. The basolateral permeation was subsequently determined using a fluorescent plate reader (Tecan Spark 10 M), with an excitation wavelength of 485 nm and emission of 530 nm.

When under flow, TEER was assessed by transferring the inserts into 24-well plates for TEER measurement, before returning to the QV600 chambers. Furthermore, tight junction formation was assessed using immunocytochemical methods as described previously.

### Mitoxantrone transport assay

PBMEC cells were grown on permeable inserts and exposed to high flow (550 µL/min) for 48 hours using the QV600. Thereafter, inserts were removed and placed into a 24-well cell culture plate. 50 µM Mitoxantrone was prepared in serum free PBMEC media containing 25 mM HEPES and added to the apical (for AB flux) or basolateral (for BA flux) compartments with sampling taking place from the opposite compartment, which contained serum free PBMEC media containing 25 mM HEPES.

Samples were taken at intervals between 15–90 minutes and replaced with equal amount fresh warm serum free PBMEC media containing 25 mM HEPES. Mitoxantrone concentrations were analysed using a fluorescent plate reader (Tecan Spark 10 M) at an excitation wavelength of 488 nm and emission wavelength of 670 nm.

The apparent permeability (P_app_) was calculated using the equation below,

$${P}_{app}=\frac{dQ/dt}{{C}_{0}\,\times \,A}$$

where dQ/dt the amount of drug permeated per unit time, calculated from the regression line of time points of sampling, C_0_ the initial drug concentration in the donor compartment and A (cm^2^) is the insert surface area (0.33 cm^2^).

### Statistical analysis

All data is presented as mean ± standard deviation, with experiments being conducted in at least 3 replicate independent experiment unless otherwise stated. Where appropriate, statistical analyses was performed in Graphpad Prism (La Jolla, California, USA), with *t*-tests used to determine differences between the mean values. A significance p-value of <0.05 was considered as statistically significant
